# Metabolic activation of mitochondria in glioma stem cells promotes cancer development through a reactive oxygen species-mediated mechanism

**DOI:** 10.1186/s13287-015-0174-2

**Published:** 2015-10-15

**Authors:** Shuqiang Yuan, Yunxin Lu, Jing Yang, Gang Chen, Sangbae Kim, Li Feng, Marcia Ogasawara, Naima Hammoudi, Weiqin Lu, Hui Zhang, Jinyun Liu, Howard Colman, Ju-Seog Lee, Xiao-Nan Li, Rui-hua Xu, Peng Huang, Feng Wang

**Affiliations:** Sun Yat-sen University Cancer Center; State Key Laboratory of Oncology in South China; Collaborative Innovation Center for Cancer Medicine, 651 E Dongfeng Road, Guangzhou, Guangdong 510060 China; Department of Translational Molecular Pathology, The University of Texas MD Anderson Cancer Center, Houston, TX 77054 USA; Department of Systems Biology, The University of Texas MD Anderson Cancer Center, Houston, TX 77054 USA; Department of Neuro-Oncology, University of Utah, Salt Lake City, UT USA; Laboratory of Molecular Neuro-oncology, Texas Children’s Cancer Center, Baylor College of Medicine, Houston, TX 77030 USA

## Abstract

**Introduction:**

Cancer stem cells (CSCs) possess characteristics associated with normal stem cells, specifically the abilities to renew themselves and to give rise to all cell types (differentiation). It is assumed that induction of differentiation in CSCs would reduce their ability to form tumors. What triggers CSC differentiation and the role of “differentiation” in tumorigenesis remain elusive.

**Methods:**

Glioma stem cell (GSC) lines and subcutaneous as well as orthotopic xenografts established from fresh surgical specimens of glioblastoma multiforme were used.

**Results:**

Exposure of GSCs to serum activates mitochondrial respiration and causes an increase in mitochondrial reactive oxygen species (ROS) as well as oxidative stress responses, leading to the appearance of differentiation morphology and a deceased expression of CSC markers. Chemical perturbation of the mitochondrial electron transport chain causes ROS increase and further downregulation of stem cell markers, while antioxidant N-acetyl-cysteine reduces ROS and suppresses the differentiation of GSCs. Surprisingly, the serum-induced differentiated GSCs exhibit greater ability to form tumor in both orthotopic and subcutaneous xenograft models, which can be suppressed by N-acetyl-cysteine. Mitochondrial ROS from the serum-stimulated cells triggered the activation of nuclear factor-kappa-B (NFκB) pathway, which is a potential mechanism for the promotion of tumorigenesis.

**Conclusion:**

This study suggests that ROS generated from active mitochondrial respiration in the presence of serum is critical in CSCs activation, which promotes tumor development *in vivo*.

**Electronic supplementary material:**

The online version of this article (doi:10.1186/s13287-015-0174-2) contains supplementary material, which is available to authorized users.

## Introduction

Recent studies indicate the existence of cancer stem cells (CSCs) in various types of cancers, including leukemia and solid tumors [1, 2]. Similar to normal stem cells, CSCs are able to self-renew and to generate the downstream progeny. Although CSCs constitute a very small fraction of the total cancer cells in the tumor bulk, this special subpopulation of malignant cells is thought to play a major role in cancer initiation and development and may be a key cause of resistance to chemotherapy and radiotherapy, leading to persistence of residual disease and cancer recurrence [3]. This phenomenon is due in part to the unique biological properties of CSCs, including high capacity of DNA repair, high expression of certain ATP-dependent drug exporting pumps, high levels of glutathione synthesis, and high expression of cell survival factors [4–6]. A detailed understanding of factors that affect the ability of CSCs to maintain their self-renewal and promote disease progression is important for developing new strategies to effectively kill CSCs.

Mounting evidence suggests that the tissue microenvironment may profoundly affect the biological properties and the fates of stem cells and CSCs [7]. *In vivo*, normal stem cells or CSCs reside in special tissue locations known as stem cell niches, which are thought to provide the microenvironment important for the maintenance of their stemness [8]. Although the exact nature of the stem cell niches remains to be defined, it is known that low oxygen and proper levels of certain growth factors such as epidermal growth factor (EGF) and basic fibroblast growth factor (bFGF) are important to maintain the stemness of the cells [9]. Brain CSCs have been found in perivascular niches [8, 10]. Increasing the endothelial cells or blood vessels in orthotopic brain tumor xenografts enhances self-renewal of CSCs and accelerates the initiation and growth of tumors [10]. However, exposure of CSCs to serum *in vitro* usually induces differentiation and presumably may compromise their self-renewal ability [11, 12]. CSCs cultured in serum-free media seem to closely mimic the genotype and gene expression profiles of their primary tumors *in vivo* than do CSCs cultured in standard serum-containing medium [9]. Although the ability of serum to induce apparent differentiation of CSCs has been known for a long time, the underlying mechanisms remain largely unknown. It is also unclear whether exposure of CSCs to serum negatively or positively affects their ability to form tumor *in vivo*.

Reactive oxygen species (ROS) are known to play a role in affecting the fates of normal stem cells [13, 14]. Elevated ROS has been observed to induce differentiation of embryonic stem cells into cardiovascular and mesendodermal cells [7, 15]. The neural stem cells and hematopoietic stem cells contain lower levels of ROS than their mature progeny, whereas increased ROS levels are associated with lowered self-renewal capacity, increased cell cycling, and reduced viability [16–18]. Previous study showed that breast CSCs might have high ROS-scavenging capacity and contain lower cellular ROS compared with the corresponding non-tumorigenic cells [6]. A recent study suggests that ROS might affect the differentiation state of CSC by activation of p38 MAPK [19]. However, the role of ROS in serum-induce differentiation of CSCs and their physiological relevance in tumor development *in vivo* remain largely unclear. The present study was designed to investigate these important questions. We showed that serum could activate mitochondrial respiration and promote generation of mitochondrial ROS, leading to apparent loss of certain stem cell markers and lower ability to form neurospheres. However, despite these seemingly differentiation phenotypes *in vitro*, the serum-induced glioma stem cells exhibited greater capacity to form tumor *in vivo*. Our study revealed a novel role of mitochondrial ROS in serum activation of CSCs to produce the downstream progeny and promote tumor development *in vivo*. The regulation of this redox signaling mechanism has potential implications in developing new strategies to target CSCs.

## Methods

### Cell lines and cell culture

GSC11, GSC23, and GBM3752 cell lines were originally established from fresh surgical specimens of glioblastoma multiforme at the University of Texas MD Anderson Cancer Center [20, 21]. GSC11 and GSC23 were maintained in Dulbecco’s modified Eagle’s medium with nutrient mixture F-12 (DMEM/F12) (Mediatech Inc., Manassas, VA, USA) supplemented with B27 (Invitrogen, Carlsbad, CA, USA), 20 ng/ml epidermal growth factor (Miltenyi Biotec, Auburn, CA, USA), 20 ng/ml of basic fibroblast growth factor (Miltenyi Biotec), and 2 mM L-glutamine (Mediatech Inc.) without serum (designated as “stem cell medium”). Cells were cultured in a humidified incubator maintained at 37 °C with 5 % CO_2_. GBM3752 cells were obtained from GBM patients undergoing surgery at Texas Children’s Hospital and maintained in severe combined immunodeficiency (SCID) mice orthotopically [22]. The cells were freshly isolated from the tumors and cultured in stem cell medium for *in vitro* study within the first five passages. For serum treatment, cells were cultured in the stem cell medium with 5 % fetal bovine serum (FBS) with or without various concentrations of N-acetyl-cysteine (NAC) (Sigma-Aldrich, St. Louis, MO, USA).

### RNA isolation, RNA microarray analyses, and reverse transcription-polymerase chain reaction

GSC11 and GSC23 cells were cultured in stem cell medium with or without serum for 1, 3, or 7 days in triplicate. Total RNA was isolated from the cells by using an RNeasy Mini kit (Qiagen Inc., Valencia, CA, USA). Sample labeling was performed with an RNA amplification kit in accordance with the conditions recommended by the manufacturer (Applied Biosystems, Foster City, CA, USA). Total RNA was reverse-transcribed by using a complementary DNA (cDNA) synthesis kit (Fermentas Inc., Glen Burnie, MD, USA). The quantitative polymerase chain reaction analyses were carried out in a 25-μl reaction mixture that contained 1 μl cDNA, 0.1 μg oligonucleotide primer pairs, 12.5 μl SYBR Green Mix (Invitrogen), and diethylpyrocarbonate-treated water. Human HT-12v3 expression beadchips containing 48,000 probes of 25,000 annotated genes were obtained from Illumina Inc. (San Diego, CA, USA). The gene expression microarray analysis was performed at the System Biology Department of the UT MD Anderson Cancer Center. Total RNA was extracted from GSC11 cells and used for labeling and hybridization to human expression beadchips in accordance with the protocols of the manufacturer. All experiments were performed in triplicate. Primary microarray data in this study are available in the National Cancer for Biotechnology Information Gene Expression Omnibus (GEO) database (GSE28220). The following primer sets were used for quantitative reverse transcription-polymerase chain reaction (RT-PCR) analysis: SOX2-sense, 5′-GCCTGGGCGCCGAGTGGA-3′; SOX2-antisense, 5′-GGGCGAGCCGTTCATGTAGGTCTG-3′); Olig2-sense, 5′-TGCGCAAGCTTTCCAAGA-3′; Olig2-antisense, 5′-CAGCGAGTTGGTGAGCATGA-3′.

### Flow cytometric analyses

Cells were dissociated into single-cell suspension by using accutase reagents (Sigma-Aldrich), stained with allophycocyanin (APC)-conjugated CD133 antibody (clone AC133 from MACS) or the control APC-IgG2b antibody (MACS) by using the conditions recommended by the manufacturer. APC fluorescence was quantitated by flow cytometry analysis. To measure intracellular ROS, cells were collected and dissociated into single-cell suspension by accutase, washed with phosphate-buffered saline (PBS) once, and resuspended in pre-warmed PBS containing freshly prepared CM-H2DCFDA (1 μM) or MitoSOX-Red (5 μM; Molecular Probes, Eugene, OR, USA). After incubation at 37 °C for 30 min (H2DCFDA) or 15 min (MitoSOX-Red), the cells were washed with PBS twice and then subjected to flow cytometric analyses.

### Immunoblots

Cultured cells were washed with cold PBS before homogenization in lysate buffer. Whole cell lysate (20 μg protein/sample) was used in Western blot analysis. Cell lysates were separated by electrophoresis on 10–12 % sodium dodecyl sulfate polyacrylamide gel electrophoresis and transferred to nitrocellulose membranes. After blocking with 5 % non-fat milk/PBS with Tween 20 for 1 h, the membranes were incubated at 4 °C overnight with primary antibodies, including mouse anti-human CD133 (Miltenyi Biotec), rabbit anti-human SOX2 (Cell Signaling Technology Inc., Danvers, MA, USA), rabbit anti-human Olig2 (Abcam, Cambridge, MA, USA), rabbit anti-human Catalase (EMD Chemicals, Gibbstown, NJ, USA), sheep anti-human SOD1 (EMD Chemicals), rabbit anti-human SOD2 (Santa Cruz Biotechnology Inc., Santa Cruz, CA, USA), and anti-mouse total OXPHOS (Abcam). The Western blot signals were detected with horseradish peroxidase-conjugated secondary antibodies. The membranes were developed by using a Pierce Supersignal West Pico Chemiluminescent Substrate (Fisher Scientific Inc., Pittsburgh, PA, USA).

### Immunofluorescence staining

Cells were fixed in 4 % formaldehyde, washed in PBS, and permeabilized for the analysis of intracellular markers (20 min, 0.25 % Triton X-100; Sigma-Aldrich). The monolayers were then incubated with a blocking solution (PBS with 5 % FBS) (45 min, room temperature), followed by incubation (overnight at 4 °C) with the primary antibodies: anti-glial fibrillary acidic protein (anti-GFAP) (Miltenyi Biotec), anti-β-III tubulin (Abcam), anti-Nestin (Abcam), and anti-O4 (Miltenyi Biotec). After extensive washing in PBS, a second incubation (1 h; 37 °C) with Alexa Fluor-488- or Alexa Fluor-547-specific anti-mouse or anti-rabbit secondary antibodies (all from Invitrogen) was performed. Cell nuclei were stained with 4′,6-diamidino-2-phenylindole (DAPI) (Sigma-Aldrich). Florescence labeling was observed by using a fluorescent microscope (Olympus, Tokyo, Japan).

### Oxygen consumption assay

Samples were dissociated into singles cells, washed with PBS once, and suspended at 4 to approximately 10 million cells per milliliter in stem cell medium. Oxygen consumption was measured in 1-ml medium by using Oxytherm equipped with a Clark-type electrode (Hansatech Instruments Ltd, Norfolk, UK) as described previously [23].

### Mouse xenografts

Subcutaneous xenografts: GSC11 cells cultured under various conditions (stem cell medium without FBS, with 5 % FBS, or with FBS and 20 mM NAC for 7 days) were collected, treated with accutase to make single-cell suspension, and inoculated into the right flank of nude mice (2 × 10^6^ cells per mouse). The mice were euthanized when the tumor diameter was greater than 1.5 cm. For orthotopic xenograft inoculation, GBM3752 cells were first cultured in stem cell medium with or without serum (5 % FBS). The cells were maintained *in vitro* under these two conditions for 60 passages. Cells were collected and inoculated intracranially into the brains of SCID mice (1 × 10^4^ cells per mouse). The mice were euthanized when they developed signs of neurological deficit and became moribund. All experiments of the present study were performed in accordance with human protocols approved by the Institutional Review Board at UT MD Anderson Cancer Center and Baylor College of Medicine as well as animal protocols (ACUF 11-98-08136, AN-4548) approved by the Institutional Animal Care and Use Committee of Baylor College of Medicine. Signed informed consent was obtained from all patients or their legal guardians prior to sample acquisition.

## Results and Discussion

### Induction of apparent differentiation of GSCs by serum and association with ROS stress responses

**Fig. 1:**
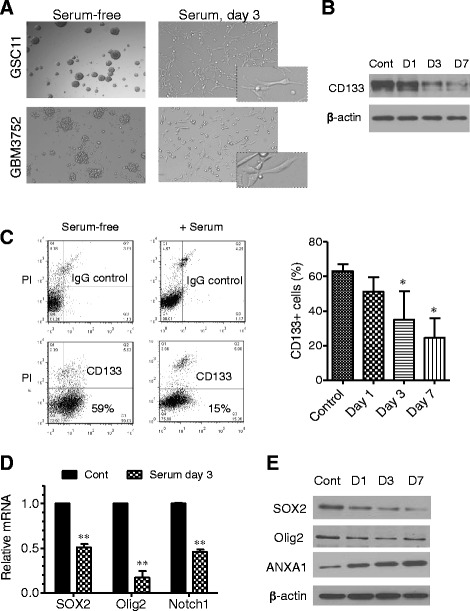
Effect of serum on neurosphere formation and the expression of stem cell markers in glioblastoma stem cells. **a** Glioblastoma stem cells (GSC11 and GBM3752) formed neurospheres in serum-free medium supplemented with epidermal growth factor and basic fibroblast growth factor. Exposure of the cells to serum (5 % FBS) for 3 days led to a loss of neurosphere formation in both clones. **b** Western blot analysis of CD133 in GSC11 cells before and after exposure to serum for 1, 3, and 7 days. **c** Flow cytometry analysis of CD133 expression in GSC11 cells before and after exposure to serum for 7 days. The *right panel* shows quantitation of the percentage of CD133^+^ cells before and after GSC11 cells were exposed to serum for 1, 3, and 7 days; *P < 0.05. **d** Expression of SOX2, Olig2, and Notch1 mRNA in GSC11 cells before and after exposure to serum for 3 days. Expression of mRNA was measured by quantitative reverse transcription-polymerase chain reaction. **P < 0.001. **e** Effect of serum on protein expression of stem cell markers SOX2 and Olig2 and differentiation marker ANXA1. GSC11 cells were exposed to 5 % FBS for 1, 3, and 7 days as indicated. SOX2, Olig2, ANXA1 were detected by Western blot analysis. *Cont* control, *D* day, *FBS* fetal bovine serum, *GBM* glioblastoma multiforme, *GSC* glioma stem cell, *PI*

Both established glioma stem cell lines and primary glioma cells isolated from fresh tumor tissues were used in our study. GSC11 and GSC23 are two glioblastoma stem cell lines originally derived from glioblastoma multiforme (GBM) surgical specimens and exhibit the *in vitro* stem cell characteristics of extensive self-renewal, the ability to differentiate to neurons and astrocytes, and the ability to initiate tumor *in vivo*. GSC11 and GSC23 cells were maintained in serum-free stem cell culture medium as described previously (He, 2010 #274) [24]. An orthotopic xenograft model (GBM3752) that preserves glioblastoma stem cells was originally established by directly inoculating primary tumor cells from fresh GBM specimen into the right cerebellum of SCID mice brain [22]. The xenograft tumor cells preserve tumorigenicity, multi-lineage differentiation, and CD133^+^ expression after being subtransplanted in mice brain. GBM3752 cells were prepared freshly from tumors (maintained in SCID) mice for *in vitro* study. As shown in Fig. 1, GSC11 and GBM3752 cells grew well in stem cell medium containing EGF and bFGF without serum and exhibited the morphology of stem-like neurospheres (Fig. 1a) with high expression of CD133 (Fig. 1b, c). The addition of serum (% FBS) to the culture medium caused a significant change in cell morphology, manifested by a loss of neurosphere formation and the appearance of differentiated cells attaching to the culture dish (Fig. 1a). This was accompanied by a substantial decrease of CD133 expression in a time-dependent manner (Fig. 1b, c) and a decrease of Nestin (Additional file 1: Figure S1). Quantitative RT-PCR and Western blot analyses revealed a significant decrease in expression of Sox2 and Olig2, two transcription factors known to regulate neural stem cells and neural progenitor cells (Fig. 1d, e). The expression of Notch1, a molecule important for promoting neural stem cell function [25], was also downregulated (Fig. 1d). In contrast, the expression of differentiation markers, including GFAP, β-III tubulin, O4 (Additional file 1: Figure S1), and ANXA1, were increased after serum exposure (Additional file 1: Figure S1 and Fig. 1e). Similar results were observed in the third cell line GSC23 (Additional file 1: Figure S2). Surprisingly, this apparent differentiation induced by serum did not result in a decrease in tumorigenesis, and as will be described below, the glioma stem cells were activated by serum exposure (see *in vivo* study below).

To investigate the molecular events and potential alterations in the signaling pathways of GSCs in response to serum induction, we treated GSC11 cells with 5 % FBS for 1, 3, and 7 days in triplicate cultures, and RNA was isolated from each sample for determination of gene expression profiles using microarray analyses. As shown in Additional file 1: Figure S3A, clustering analysis of gene expression profiles revealed that the serum-treated GSC11 cells exhibited gene expression profiles clearly distinct from that of the GSC11 cells cultured in serum-free medium. There was a further shift of gene expression profiles as the time of serum exposure prolonged. The fact that the three separated samples of the same time point (biological triplicate) displayed similar gene expression patterns and clustered in the same group demonstrated the high reproducibility of this experimental system. Using the Ingenuity pathway analysis, we found that the oxidative stress response pathway was induced by serum most significantly (*P* = 0.0005) at all time points tested. Additional file 1: Figure S3B shows the genes involved in oxidative stress response identified by this analysis in GSC11 cells. Among these genes, SOD2, catalase, NQO1, peroxiredoxin 1, thioredoxin reductase 1, and glutamate-cysteine ligase are involved in ROS scavenging. These results suggested that the homeostasis of reduction/oxidation (redox) balance might have been disrupted in the serum-induced GSCs.

### Induction of mitochondrial ROS generation in glioma stem cells by serum through activation of electron transport chain

**Fig. 2:**
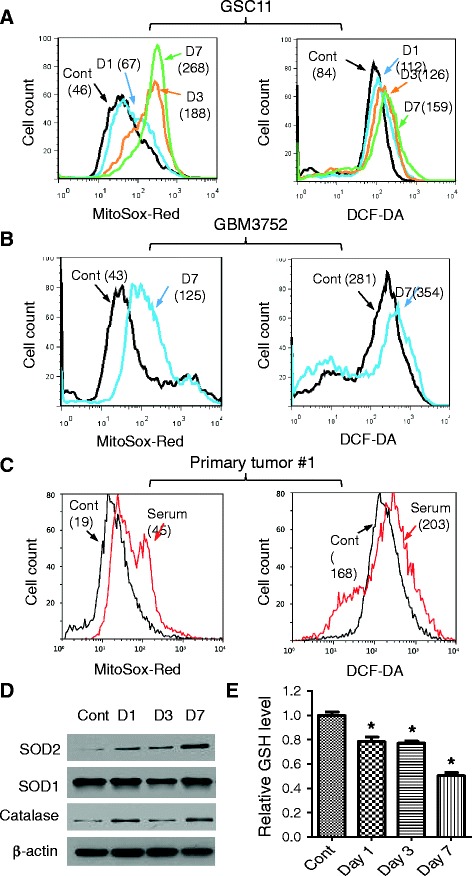
Induction of mitochondrial O_2_^−^ generation and oxidative stress response in glioblastoma stem cells. **a** Effect of serum on mitochondrial O_2_^−^ and total cellular ROS in GSC11 cells. Cells were incubated without or with 5 % FBS for 1, 3, or 7 days (D1, D3, D7). Mitochondrial O_2_^−^ was measured by flow cytometry analysis after cells were stained with MitoSOX-Red, and total cellular ROS were detected by using CM-H2-DCFDA staining followed by flow cytometry analysis. The numbers in parentheses indicate the median fluorescent intensity. **b** Effect of serum exposure (1–7 days) on mitochondrial O_2_^−^ and total cellular ROS in GBM3752 cells. **c** Effect of serum on mitochondrial O_2_^−^ and total cellular ROS in primary tumor cells isolated from fresh GBM tumor tissue. The freshly isolated tumor cells were divided into two portions for incubation in stem cell medium without or with serum (5 % FBS) for 7 days, and mitochondrial O_2_^−^ and total cellular ROS were then measured. The shaded curve shows the background (Bkg) fluorescent without dye. **d** Western blot analysis of SOD1, SOD2, and catalase in GSC11 cells before and after exposure to serum for 1, 3, and 7 days. **e** Cellular glutathione in GSC11 cells cultured in stem cell medium without or with serum for 1, 3, and 7 days. *P < 0.05. *Cont* control, *DCF-DA* 5-(and-6)-chloromethyl-2,7-dichlorodihydrofluorescein diacetate acetyl ester, *FBS* fetal bovine serum, *GBM* glioblastoma multiforme, *GSH* glutathione, *GSC* glioma stem cell, *O*_*2*_^*−*^ superoxide, *ROS* reactive oxygen species, *SOD* cytosolic superoxide dismutase

The observations that exposure of GSCs to serum caused consistent oxidative stress response in all tested time points prompted us to explore possible changes in cellular redox status. Since mitochondria are major sites of ROS production, we used MitoSOX-Red to detect mitochondrial superoxide (O_2_^−^) and 5-(and-6)-chloromethyl-2,7-dichlorodihydrofluorescein diacetate acetyl ester (DCF-DA) to measure total cellular hydrogen peroxide (H_2_O_2_) and other ROS. The results showed that that serum induced a substantial increase of mitochondrial O_2_^−^ in a time-dependent manner, with an increase of the median value from 46 units in control cells (serum-free) to 67, 188, and 268 units on days 1, 3, and 7 after serum exposure, respectively (Fig. 2a). Interestingly, total cellular ROS (as measured by DCF-DA) also showed a moderate increase, from 84 units in the control to 112, 126, and 159 units on days 1, 3, and 7, respectively (Fig. 2a). Similar results were observed in another glioblastoma stem cell line GSC23 (Additional file 1: Figure S4). Exposure of GSC23 cells to serum led to a 5- and 11-fold increase of mitochondrial O_2_^−^ on days 3 and 7, respectively, and the total cellular ROS detected by DCF-DA also moderately increased (Additional file 1: Figure S4A).

We then used two types of fresh glioma cells to further confirm the above observations. First, the stem-like GBM3752 cells [22] were obtained freshly from tumor xenografts and divided into two portions; one portion was cultured in serum-free stem cell medium and the other portion was cultured in serum-containing medium. After 7 days, the mitochondrial ROS and total cellular ROS in each culture condition were measured by MitoSOX and DC-FDA. As shown in Fig. 2b, a 3-fold increase in mitochondrial O_2_^−^ and a moderate increase (26 %) in total cellular ROS were observed, consistent with that seen in GSC11 and GSC23 cells. Furthermore, this pattern of redox alterations was consistently observed in primary glioma cells isolated from fresh GBM tumor tissues (Fig. 2c and Additional file 1: Figure S4B), suggesting that the induction of mitochondrial O_2_^−^ generation might be a highly consistent event in serum-induced changes in GSCs.

To test whether the increase in mitochondrial O_2_^−^ and cellular ROS induced by serum in GSCs might cause stress response, we used Western blotting to analyze the expression of antioxidant molecules before and after serum exposure. As shown in Fig. 2d, there was a time-dependent increase in expression of SOD2, a mitochondrial superoxide dismutase that converts O_2_^−^ to H_2_O_2_. Interestingly, the cytosolic superoxide dismutase (SOD1) did not exhibit significant change after GSC11 and GSC23 were exposed to serum (Fig. 2d, Additional file 1: Figure S4C), suggesting that the main source of ROS stress might be mainly from mitochondria and was consistent with the increase in mitochondrial O_2_^−^ shown in Fig. 2a-c. The expression of catalase, an enzyme that converts cellular H_2_O_2_ to water and oxygen, increased after serum incubation (Fig. 2d, Additional file 1: Figure S4C). Cellular glutathione (GSH), a major endogenous antioxidant, decreased after serum exposure in GSC11 cells (Fig. 2e) and G23 cells (Additional file 1: Figure S4D), reflecting a consumption of this antioxidant. These data together suggest that the increase in SOD2 expression might be a stress response to elevated mitochondrial O_2_^−^ generation induced by serum. SOD2 converted O_2_^−^ to H_2_O_2_, which was then able to pass the mitochondrial membranes to cytosol, where it was converted to O_2_ and H_2_O by catalase or neutralized by GSH, resulting in only a moderate increase of overall cellular ROS and a decrease in GSH.

**Fig. 3:**
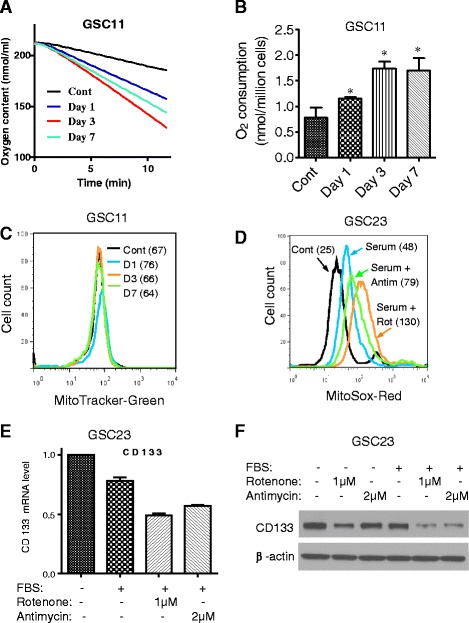
Novel role of mitochondrial activation and ROS generation in activation of glioblastoma stem cells. **a** Comparison of oxygen consumption in GSC11 cells before and after exposure to serum for 1, 3, and 7 days. Oxygen consumption was measured by using an Oxytherm system as described in Methods. **b** Quantitative analysis of oxygen consumption in GSC11 cells exposed to serum for 1, 3, and 7 days. *P < 0.01. **c** Serum did not induce increase of mitochondrial mass in GSC11 cells, measured by MitoTracker-Green. **d** GSC23 cells were cultured in stem cell medium without or with serum (5 % FBS) in the presence or absence of 1 μM rotenone (ETC. complex I inhibitor) or 2 μM antimycin (complex III inhibitor), and mitochondrial O_2_^−^ was measured by flow cytometric analysis after cells were stained with MitoSOX-Red. **e** Effect of serum and ETC. inhibitors on CD133 expression. GSC23 cells were cultured in stem cell medium in the presence or absence of serum (5 % FBS), rotenone (1 μM), or antimycin (2 μM). CD133 mRNA expression levels were measured by quantitative reverse transcription-polymerase chain reaction. **f** GSC23 cells were cultured in stem cell medium in the presence or absence of serum (5 % FBS), rotenone (1 μM), or antimycin (2 μM). CD133 protein expression was measured by Western blot. *Cont* control, *ETC*. electron transport chain, *FBS* fetal bovine serum, *GSC* glioma stem cell, *O*_*2*_^*−*^ superoxide

Since mitochondrial O_2_^−^ is generated mainly during respiration because of the release of electrons from complexes I and III of the electron transport chain, we speculated that the increased mitochondrial O_2_^−^ might be a result of active mitochondrial respiration induced by serum and not a consequence of a slower O_2_^−^ elimination since SOD2 expression was increased. To test this possibility, we measured oxygen consumption in GSCs as an indicator of mitochondrial respiration. As shown in Fig. 3a and b, exposure of GSC11 cells to serum led to a time-dependent increase of oxygen consumption, with approximately a 100 % increase by day 3. Interestingly, measurement of mitochondrial mass by using MitoTracker Green as well as mitochondria electron transport chain (ETC.) and the ATP synthase complex antibodies showed that serum did not cause any significant change in mitochondrial mass and complexes (Fig. 3c and Additional file 1: Figure S5), suggesting that the increase in respiration was mainly a functional activation of the pre-existing mitochondria. GBM3752 cells from freshly dissected orthotopic tumor xenografts were cultured in either serum-free medium or serum-containing medium for 7 days. A significantly higher oxygen consumption was observed in GBM3752 cells cultured with serum (Additional file 1: Figure S6A) without any significant changes in mitochondrial mass (Additional file 1: Figure S6B). The increase in respiration without increase of mitochondrial mass was also consistently observed in GSC23 cells (Additional file 1: Figure S6C). Despite the increase of mitochondrial respiration, cells at G_0_/G_1_ phase were decreased only on day 1 compared with cells cultured in serum-free medium (Additional file 1: Figure S7). Cells at G_2_/M phase were not changed.

### Important role of mitochondrial ROS in mediating the serum effect on glioma stem cells

To evaluate the role of activation of mitochondrial respiration and ROS generation in serum-induced apparent differentiation of GSCs, we first used ETC. complex I inhibitor rotenone and complex III inhibitor antimycin to inhibit mitochondrial respiration in glioma stem cells and then tested whether this affected the ability of serum to induce apparent differentiation in GSCs. The results showed that both ETC. inhibitors disrupted mitochondrial respiration and caused a further increase of mitochondrial O_2_^−^ in the presence of serum (Fig. 3d), but neither of them prevented the serum-induced GSCs from attaching to the flasks and exhibiting apparent differentiation morphology (data not shown). In fact, adding rotenone or antimycin even caused a further decrease of CD133 at both mRNA (Fig. 3e) and protein levels (Fig. 3f).

**Fig. 4:**
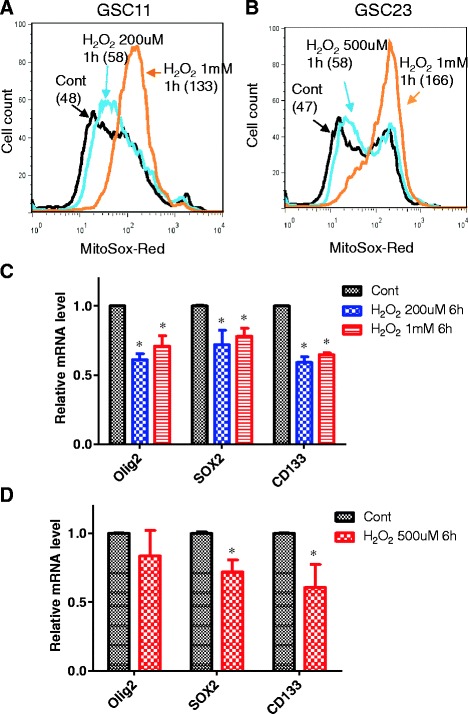
Exogenous H_2_O_2_ suppressed the expression of Olig2, SOX2, and CD133 in GSCs. **a** Exogenous H_2_O_2_ caused an increase in mitochondrial O_2_^−^ in GSC11 cells. Cells were treated with 200 μM or 1 mM of H_2_O_2_ for 1 h, and mitochondrial O_2_^−^ was measured by flow cytometric analysis. **b** Exogenous H_2_O_2_ caused an increase in mitochondrial O_2_^−^ in GSC23 cells. Cells were treated with 500 μM or 1 mM of H_2_O_2_ for 1 h, and mitochondrial O_2_^−^ was then measured by flow cytometric analysis. **c** GSC11 cells were treated with 200 μM or 1 mM H_2_O_2_ for 6 h with addition of H_2_O_2_ at 1-h intervals. Expression of SOX2, Olig2, and CD133 mRNA in GSC11 cells was measured by quantitative reverse transcription-polymerase chain reaction. *P < 0.05. **d** Effect of exogenous H_2_O_2_ on the expression of SOX2, Olig2, and CD133 in GSC23 cells under the same conditions as in (c). *P < 0.05. *Cont* control, *GSC* glioma stem cell, *H*_*2*_ *O*_*2*_ hydrogen peroxide, *O*_*2*_^*−*^ superoxide

Considering the observations that exposure of GSCs to serum caused an increase in mitochondrial respiration and O_2_^−^ generation but inhibition of mitochondria respiration by ETC. inhibitors (rotenone and antimycin) did not prevent serum to induce changes in GSCs, we speculate it was the increase in mitochondrial ROS generation, not the respiration *per se*, that plays a key role in mediating the serum effect on GSCs. To test this possibility, we used exogenous H_2_O_2_ to cause a level of increase in mitochondrial ROS comparable to that caused by serum in GSC11 and GSC23 cells (Fig. 4a, b). Interestingly, a short-term treatment of GSCs with such exogenous H_2_O_2_ for 6 h led to a significant decrease of SOX2, Olig2, and CD133 mRNA expression in GSC11 cells (Fig. 4c) and GSC23 cells (Fig. 4d), similar to those observed in serum-induced cells.

**Fig. 5:**
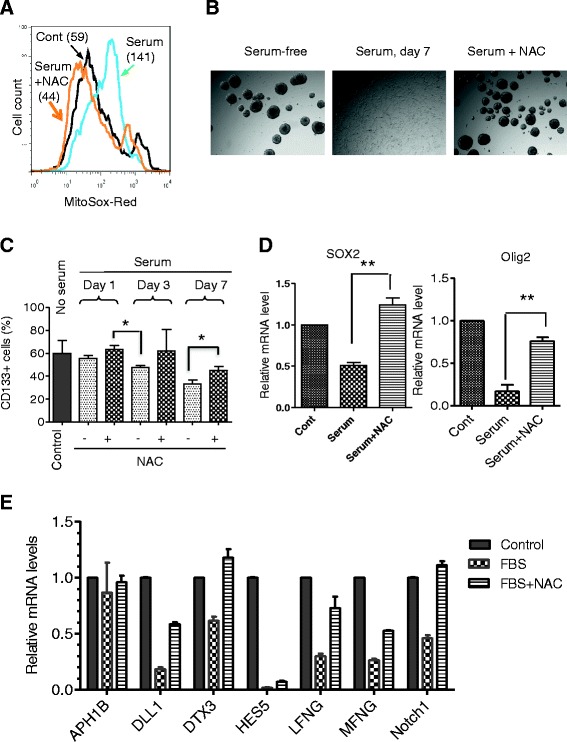
Effect of antioxidant NAC on serum-induced ROS generation and the expression of stem cell markers in GSCs. **a** Effect of NAC on mitochondrial O_2_^−^ levels in GSC11 cells exposed to serum. Cells were cultured in stem cell medium with or without serum (5 % FBS) in the presence or absence of 20 mM NAC for 7 days. Mitochondrial O_2_^−^ was measured by using MitoSOX-Red. **b** Comparison of neurosphere formation in GSC11 cells cultured in serum-free or serum-containing medium in the presence and absence of 20 mM NAC for 7 days. **c** NAC suppressed serum-induced loss of CD133 expression. GSC23 cells were exposed to serum for 1, 3, or 7 days in the presence or absence of 20 mM NAC as indicated, and CD133-positive cells were quantitated by flow cytometry analysis. *P < 0.05. **d** GSC11 cells were exposed to serum (5 % FBS) for 3 days in the presence and absence of 20 mM NAC. The expression of SOX2 and Olig2 mRNA was measured by quantitative RT-PCR. **P < 0.001. **e** NAC suppressed serum-induced downregulation of the Notch pathway. GSC11 cells were incubated without or with serum (5 % FBS) for 3 days in the presence or absence of 20 mM NAC. RNA was isolated from each sample, and the expression of molecules involved in Notch signaling was measure by quantitative RT-PCR. *FBS* fetal bovine serum, *GSC* glioma stem cell, *NAC* N-acetyl-cysteine, *O*_*2*_^*−*^ superoxide, *ROS* reactive oxygen species, *RT-PCR* reverse transcription-polymerase chain reaction

To further validate this novel role of ROS, we used NAC, a precursor for glutathione synthesis with potent antioxidant property to reduce ROS stress, to test whether it could prevent the effect of serum on GSCs. As shown in Fig. 5a, incubation of GSC11 cells with serum for 7 days caused a significant increase of mitochondrial O_2_^−^, and the presence of NAC effectively suppressed such ROS increase. Importantly, NAC also prevented serum-induced loss of ability to form stem-like neurospheres (Fig. 5b) and partially preserved the expression of CD133 in serum-induced cells (Fig. 5c). These results suggest that the increase in mitochondrial ROS generation might play an important role in mediating the serum effect on GSCs. Since there was a significant decrease in expression of SOX2, Olig2, and Notch1 in serum-induced GSCs, we tested whether NAC might also suppress this serum effect. As shown in Fig. 5d, quantitative RT-PCR revealed that the addition of NAC to the serum-treated GSC11 cells largely blocked the decrease of SOX2 and Olig2 expression, suggesting that the expression of these two molecules might be redox-sensitive. Similar results were observed in GSC23 (Additional file 1: Figure S8). Furthermore, gene expression analysis of molecules involved in the Notch pathway revealed that serum caused a significant decrease in the expression of Notch1, MFNG, LFNG, HESs, DTX3, and DLL1 (Fig. 5e). Consistently, the presence of antioxidant NAC largely prevented the downregulation of the Notch-related genes (Fig. 5e), again suggesting the important role of ROS and redox signaling in regulation of GSCs.

### Serum induction of GSCs promotes tumorigenesis *in vivo*

**Fig. 6:**
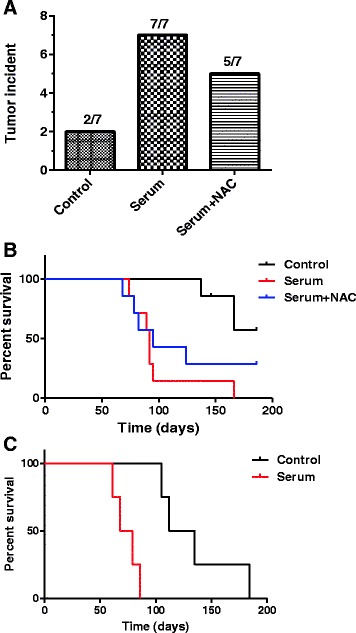
Serum promotes *in vivo* tumorigenesis of glioblastoma stem cells. **a** GSC11 cells were cultured in serum-free medium or serum-containing medium in the presence or absence of 20 mM NAC for 7 days. Equal numbers of cells (2 × 10^6^) from each sample were inoculated subcutaneously into the right flank of nude mice (seven mice per group). The numbers of mice that developed tumors in each group are shown. **b** Survival curves for the mice in the three groups of mice described in (a). **c** GBM3752 cells were isolated from orthotopic tumor xenografts cultured in stem cell medium with or without serum as described in Methods. Equal numbers of cells (10,000 per injection) were inoculated into non-obese diabetic/severe combined immunodeficiency mice intracranially. The mice were observed for survival and euthanized when they developed signs of neurological deficit and became moribund. *NAC* N-acetyl-cysteine

Because exposure of GSCs to serum caused the cells to exhibit apparent differentiation morphology and a decrease in neurosphere formation, we used two *in vivo* models to test whether serum induction of such changes might alter the ability of GSCs to form tumors *in vivo*. First, GSC11 cells were incubated with or without serum for 7 days, and the same numbers (2 × 10^6^) of the control cells or serum-induced cells were inoculated subcutaneously into the right flank of nude mice. Under these subcutaneous inoculation conditions, only two out of seven of the mice inoculated with the control GSC11 cells (serum-free) form tumors, and surprisingly seven out of seven mice inoculated with the serum-induced GSC11 cells developed tumor (Fig. 6a), suggesting that exposure of GSC11 cells to serum promotes their tumorigenesis. Interestingly, five out of seven mice inoculated with GSC11 cells treated with serum in the presence of the antioxidant NAC formed tumors (Fig. 6a). The overall survival of the mice inoculated with serum-induced GSC11 cells was significantly shorter than that of the control mice inoculated serum-free GSC11 cells (*P* = 0.0019, Fig. 6b). No significant difference (*P* = 0.064) in overall survival was found between the control group (serum-free) and the group of mice inoculated with GSC11 cells exposed to serum in the presence of NAC (20 mM).

We also used another mouse model, orthotopic inoculation of GBM3752 cells into the SCID mice (Shu et al. [22]), to further evaluate the role of serum exposure on tumorigenesis. The same numbers (1 × 10^4^) of GBM3752 cells with or without serum exposure were inoculated into SCID mice intracranially, and the mice were observed for tumor development and survival. As shown in Fig. 6c, the overall survival of mice inoculated with serum-induced GBM3752 cells was significantly shorter than that of the mice bearing the control GBM3752 cells cultured in stem cell medium without serum (*P* = 0.0067). These results were consistent with those of the subcutaneous tumor model and suggest that serum exposure activated glioma stem cells and promoted tumor formation.

### Activation of the NFĸB survival pathway in serum-induced glioma stem cells

**Fig. 7:**
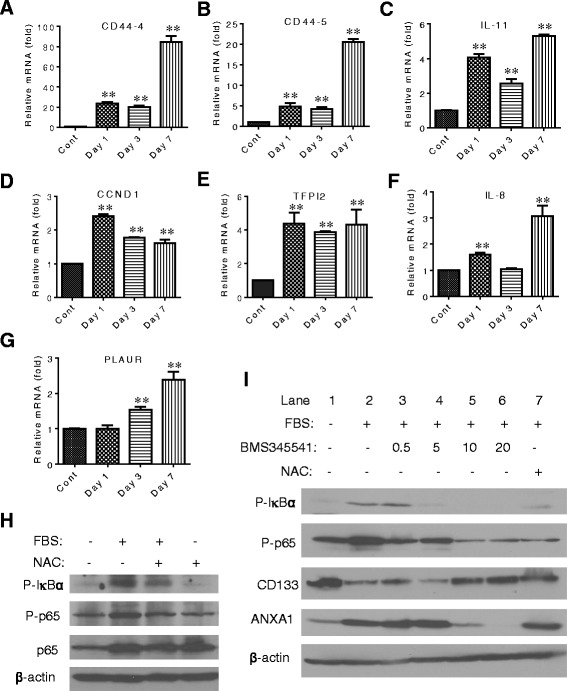
Activation of the NFĸB pathway by serum in glioblastoma stem cells is associated with ROS stress. **a**–**g** Induction of expression of genes which are downstream of NFĸB by serum exposure. GSC11 cells were cultured in serum-free medium or serum-containing medium for 1, 3, and 7 days. The relative mRNA expression levels of the target genes were analyzed by microarray assays in triplicate for each time point. **h** Antioxidant NAC suppressed serum-induced NFĸB pathway activation in GSC23 cells. Protein samples were obtained from GSC23 cells cultured in serum-free or serum-containing medium in the presence or absence of 20 mM NAC for 3 days. The expression of phospho-IKBα, phosphor-p65, total p65, and β-actin was assayed by Western blot. **i** IKK inhibitor BMS-345541 and antioxidant NAC suppressed serum-induced NFĸB activation in GSC11 cells. GSC11 cells were cultured in serum-free or serum-containing medium with the indicated concentrations of BMS345541 or 20 mM of NAC for 3 days. The expression of phospho-IKBα, phosphor-p65, CD133, ANXA1, and β-actin was measured by Western blot analysis. *CCND1* cyclin D1, *CD44-4* CD44 transcript variant 4, *CD44-5* CD44 transcript variant 5, *FBS* fetal bovine serum, *GSC* glioma stem cell, *IL-8* interleukin 8, *IL-11* interleukin 11, *NAC* N-acetyl-cysteine, *NFĸB* nuclear factor-kappa-B, *PLAUR* plasminogen activator urokinase receptor, *ROS* reactive oxygen species, *TFP1-2* tissue factor pathway inhibitor 2

To explore the possible mechanisms by which serum induction of mitochondrial ROS generation could lead to activation of GSCs and promote tumorigenesis, we further analyzed the gene expression microarray data from GSC11 cells exposed to serum for various times (Additional file 1: Figure S2) and found that the expression of multiple genes downstream of nuclear factor-kappa-B (NFκB) (including CD44, IL-8, IL-11, CCND1, TFPI2, and PLAUR) consistently increased after incubation with serum (Fig. 7a–g). Since oxidative stress is known to activate NFκB [26, 27], it is possible that *in vivo*. Indeed, incubation of GSC23 cells with serum caused a substantial increase in IκBα phosphorylation and p65 phosphorylation (Fig. 7h), two molecular events indicative of NFκB activation. Importantly, the addition of the antioxidant NAC partially suppressed the serum-induced phosphorylation of IκBα and p65, suggesting the role of ROS in mediating serum-induced NFκB activation. Similar results were observed in GSC11 cells (Fig. 7i). Serum exposure caused a significant increase in phosphorylated IκBα and p65 (Fig. 7i, lanes 1 and 2). The addition of NAC suppressed these phosphorylations (Fig. 7i, lane 7). These data together with the known function of IKK in phosphorylating IκBα and p65 suggest a possibility that serum might activate NFκB in GSCs through ROS-induced activation of IKK, a redox-sensitive molecule known to be activated by ROS [28]. To exam this possibility, we used a specific IKK inhibitor, BMS-345541, to test whether inhibition of IKK would prevent serum-induced phosphorylation of IκBα and p65. As shown in Fig. 7i, BMS-345541 at concentrations of 10–20 μM effectively suppressed serum-induced phosphorylation of IκBα and p65, associated with a preservation of CD133 expression of suppression of ANXA1. These data suggest that IKK might play an important role in mediating serum-induced activation of NFκB through a redox-sensitive mechanism.

It has been known for some time that CSCs, similar to normal stem cells, require a certain tissue microenvironment to maintain their stemness [29, 30] and that exposure of stem cells to serum *in vitro* usually induces differentiation phenotype [9]. However, the underlying mechanisms remain unclear. Although apparent differentiation phenotypes are also observed when CSCs are exposed to serum, it is unclear whether this would cause a decrease in tumorigenesis. Our study revealed a ROS-mediated mechanism by which serum induces apparent differentiation in glioma stem cells. We found that exposure of GSCs to serum resulted in activation of mitochondrial respiration, leading to an increase of oxygen consumption and high generation of mitochondrial O_2_^−^, which induced the expression of SOD2 to convert O_2_^−^ to H_2_O_2_. Owing to its relatively long half-life and ability to cross biological membranes, H_2_O_2_ has been considered a second messenger that mediates redox-sensitive signaling in cellular response to growth factors [31, 32]. The ability of H_2_O_2_ to cause oxidation of protein thiol via catalytic cysteine can alter the function of the target proteins and thus provides a mechanism for redox signaling [32]. Since O_2_^−^ has an extremely short half-life and cannot pass the mitochondrial membranes, the increased O_2_^−^ within the mitochondria could not directly function as a second messenger to affect the nuclear gene expression. As such, conversion of the mitochondrial O_2_^−^ to H_2_O_2_ by SOD2 seems important to relay the redox signal from mitochondria to cytosol and nucleus during serum-induced differentiation of GSCs. The upregulation of SOD2 in serum-treated GSCs would facilitate the conversion of O_2_^−^ to H_2_O_2_.

Although the ability of ROS to induce normal stem cell differentiation has been noticed in some experimental systems [33, 34], the exact mechanisms remain unclear. Our study found that downregulation of SOX2, Olig2, and the Notch-related molecules by ROS might be a potential mechanism. The ability of exogenous antioxidant NAC to prevent the decreased expression of these genes and to block the serum-induced differentiation phenotype supports this notion. It is possible that the expression of SOX2, Olig2, and the Notch-related molecules is regulated via a redox-sensitive mechanism, with ROS being a negative regulator. Since SOX2, Olig2, and the Notch pathway are involved in regulation of neural stem cells [35, 36], downregulation of these genes could promote apparent differentiation of GSCs and drive them to enter a process in which GSCs progress to become the downstream progeny cancer cells.

Surprisingly, we found that although incubation of GSCs with serum induced apparent differentiation morphology and caused a downregulation of certain stem cell markers, including CD133, SOX2, and Olig2, this led to an increase of tumorigenicity and a reduction of survival in mice in two different mouse models (subcutaneous and orthotopic). These new findings seem to challenge the traditional view that CSCs are responsible for cancer development and their ability to form tumor would decrease once they are induced to undergo differentiation. Interestingly, a recent study showed that tumor cell self-renewal capacity did not predict tumor growth potential *in vivo* [37]. The study by Barrett et al. showed that glioma cells with low self-renewal capacity were more tumorigenic and generate tumor more rapidly than cells with high self-renewal capacity. These observations are consistent with our findings. Furthermore, our study revealed that activation of the nuclear factor-kappa-B (NFĸB) survival pathway by ROS might be a mechanism that promotes GSC survival and enhances tumorigenesis. The role of the NFĸB pathway in normal stem cell proliferation has been implicated previously. For instance, NFĸB is activated during human embryonic stem cell (hESC) differentiation, and inhibition of NFĸB leads to a reduction of hESC proliferation and suppression of their progression toward primitive extraembryonic and embryonic lineages [38]. As such, inhibition of NFĸB may potentially prevent activation of CSCs and thus suppress tumor development. Interestingly, recent studies have shown that targeting the NFĸB pathway may be effective against CSCs [39–41]. Their studies showed that inhibition of NFĸB by using compounds such as niclosamide and disulfiram suppressed acute myeloid leukemia stem cells and breast CSCs.

The NFĸB signaling pathway plays a crucial role in cancer development and progression [42]. It can either promote or inhibit carcinogenesis, depending on the cell types and experimental conditions. The NFĸB signaling pathway is often activated by ROS through IKK phosphorylation of IĸB [43] and p65 [44]. Our study found that serum could cause the activation of NFκB, which could be blocked by the antioxidant NAC. These results suggest that the activation of NFĸB in the serum-induced cells is probably mediated by a redox-regulatory mechanism due to ROS generation in the serum-activated mitochondria. It is worth noting that deletion of NFĸBIA which encodes the NFĸB inhibitor IĸBα seems to be an oncogenic event in GBM [45]. Interestingly, a recent study showed that the NFĸB pathway was activated in glioblastoma-initiating cells (GICs) after induction of differentiation and that blockade of the NFĸB pathway could drive differentiating GSCs into senescence [40], again suggesting that activation of NFĸB is important in maintaining the proliferation of differentiating GSCs. This effect was partly mediated by reduced levels of the NFκB target gene cyclin D1. Furthermore, a novel small-molecule inhibitor of the NFκB pathway induced senescence of tumor cells in a mouse model bearing human GIC-derived tumors. These findings reveal that activation of NFκB may keep differentiating GICs from acquiring a mature postmitotic phenotype, thus allowing cell proliferation. Our study showed that, in the case of GSC exposure to serum, activation of NFĸB is likely through ROS stimulation of IKK, which promotes phosphorylation of IĸBα and p65. We found that antioxidant NAC or inhibition of IKK by BMS-345541 could effectively prevent the serum-induced NFĸB activation and loss of CD133, suggesting a novel role of ROS in driving the progression of GSCs toward downstream progeny cells to promote tumor development.

## Conclusions

In summary, our study showed that exposure of glioma stem cells to serum could stimulate mitochondrial respiration leading to increased generation of mitochondrial ROS, which activated NFκB to promote cancer cell survival and tumorigenesis. A key underlying mechanism is likely through a redox-mediated activation of IKK to phosphorylate IκBα and p65. Although the serum-induced elevation of ROS in GSCs also caused a decrease in neurosphere formation *in vitro* and a reduced expression of stem cell markers such as CD133, this apparent differentiation did not reduce the ability of the glioma stem cells to form tumor *in vivo*. Instead, the serum-induced GSCs exhibited greater tumorigenesis in both subcutaneous and orthotopic xenograft models. These new findings suggest that serum may activate glioma stem cells to progress toward the downstream cancer progenitor cells and promote tumor formation and that activation of mitochondrial respiration and ROS generation may play a key role in redox signaling during this tumorigenesis process. It is also important to note that the apparent differentiation phenotype such as neurosphere formation and CD133 expression *in vitro* observed might not necessarily predict tumorigenesis *in vivo*.

### Accession numbers

The Gene Expression Omnibus (GEO) accession number for the RNA microarray data in this study is GSE28220.

## Additional file

Additional file 1:
**Supplementary Figures.** (PDF 628 kb)

## Abbreviations

APC: Allophycocyanin
bFGF: Basic fibroblast growth factor
CSC: Cancer stem cell
DCF-DA: 5-(and-6)-chloromethyl-2,7-dichlorodihydrofluorescein diacetate acetyl ester
EGF: Epidermal growth factor
ETC.: Electron transport chain
FBS: Fetal bovine serum
GBM: Glioblastoma multiforme
GIC: Glioblastoma-initiating cell
GSC: Glioma stem cell
GSH: Glutathione
H_2_O_2_: Hydrogen peroxide
hESC: Human embryonic stem cell
NAC: N-acetyl-cysteine
NFκB: Nuclear factor-kappa-B
O_2_^−^: Superoxide
PBS: Phosphate-buffered saline
ROS: Reactive oxygen species
RT-PCR: Reverse transcription-polymerase chain reaction
SCID: Severe combined immunodeficiency
SOD1: Cytosolic superoxide dismutase
UT: University of Texas
