# Supplementary material for: Metabolic activation of mitochondria in glioma stem cells promotes cancer development through a reactive oxygen species-mediated mechanism
Source: Stem Cell Res Ther. 2015 Oct 15;6:198. doi: 10.1186/s13287-015-0174-2 (PMC4606508; doi:10.1186/s13287-015-0174-2)
Supplement: Additional file 1: — Supplementary Figures. (PDF 628 kb) [file 13287_2015_174_MOESM1_ESM.pdf]

Supplementary Figure 1

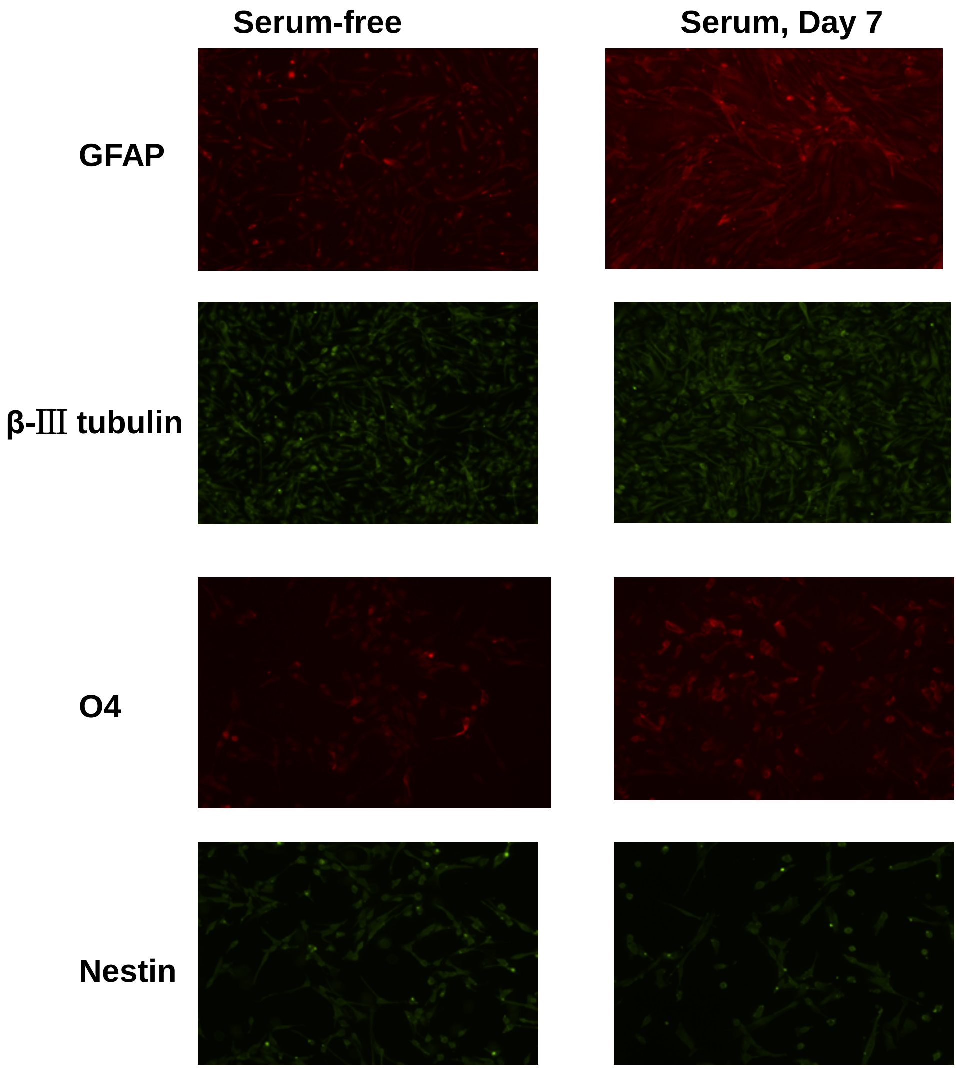

Fig S1. Effect of serum on the expression of stem cell marker and differentiation markers in GSC23 cells. Immunofluorescence staining of GFAP, β-Ⅲ tubulin, O4 and Nestin in GSC11 cells before and after exposure to 5% FBS for 7 days.

Supplementary Figure 2

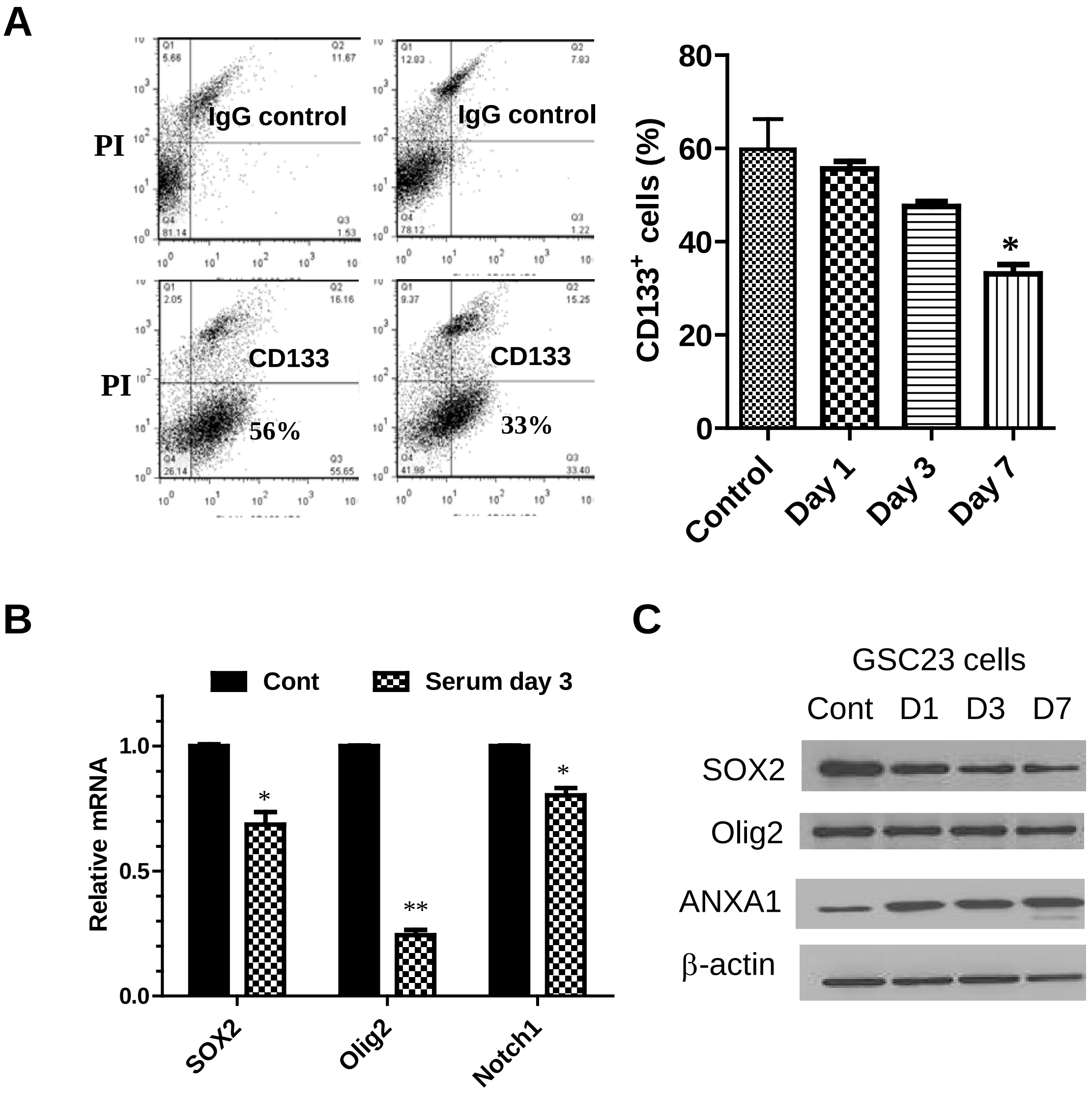

Fig S2. Effect of serum on the expression of stem cell markers in GSC23 cells. (A) Flow cytometry analysis of CD133 expression in GSC23 cells before and after exposure to serum for 7 days. Quantitation of the percentage of $CD133^+$ cells before and after GSC23 cells were exposed to serum for various times (1, 3, and 7 days) is shown on the right panel. *. P<0.05. (B) Expression of SOX2, Olig2, and notch1 mRNA in GSC23 cells before and after exposure to serum for 3 days. Expression of mRNA was measured by quantitative real-time PCR. *, P<0.05; **, p<0.001. (C) Effect of serum exposure on protein expression of stem cell markers SOX2 and Olig2 and differentiation marker ANXA1 detected by western blot analysis. GSC23 cells were exposed to 5% FBS for 1, 3, and 7 days, and gene expression was analyzed by western blotting.

Supplementary Figure 3

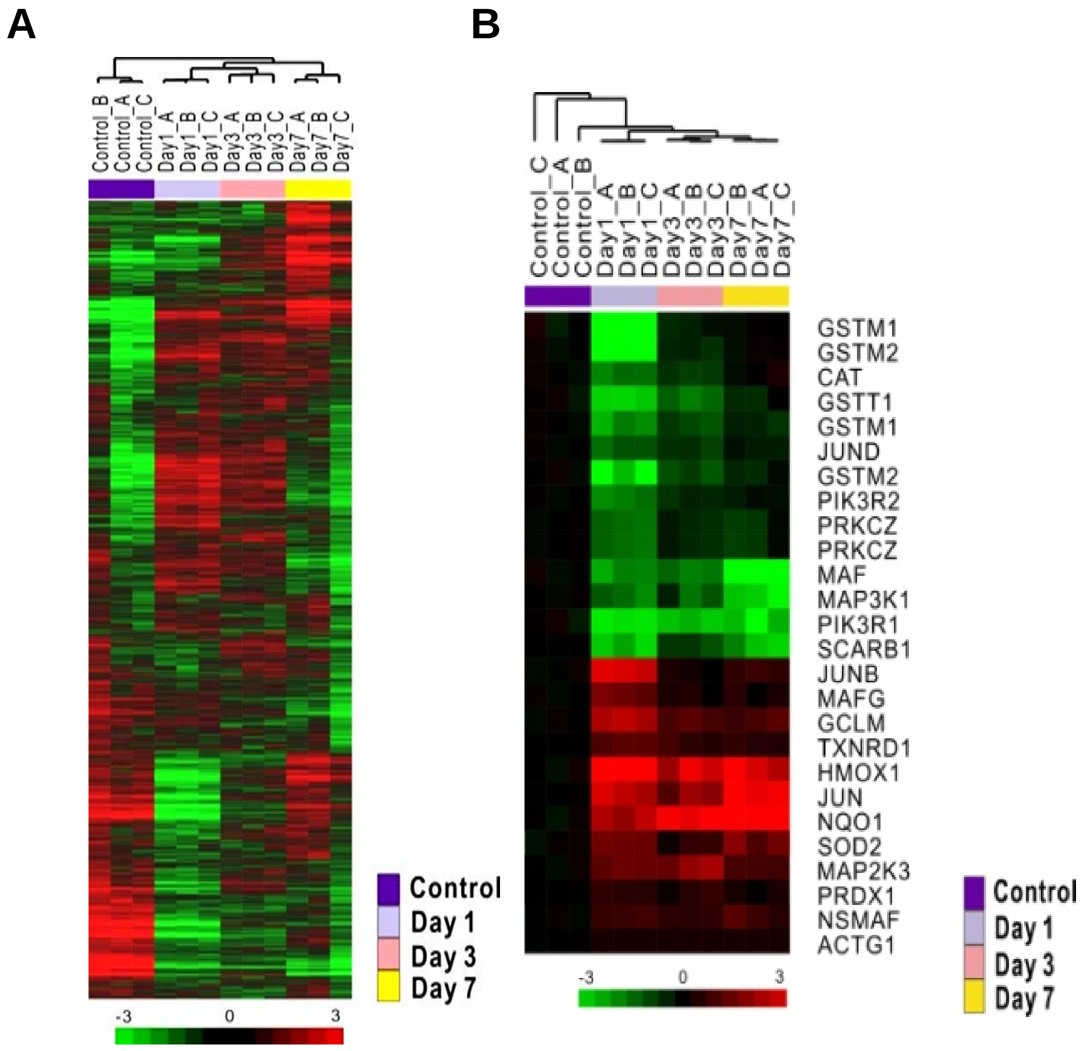

Fig S3. Analyses of gene expression profiles in glioblastoma stem cells before and after exposure to serum. (A) Comparison of gene expression patterns in GSC11 cells exposed to serum for various times. GSC11 cells maintained in serum-free stem cell medium were incubated with 5% FBS for 1, 3, and 7 days (each time point contained triplicate culture flasks A-C). After proper variance filtering, the gene expression data were used for unsupervised hierarchical clustering analysis with similarity metric of uncentered correlation. The intensity of red and green color represents the degree of high and low expression, respectively, as indicated in the log2-transformed scale bar. (B) List of genes associated with the Nrf2-mediated oxidative stress response pathway. Genes with expression showing significant differences between control (serum-free) and serum-induced conditions in GSC11 cells were identified by two-tail t-test ($p < 0.001$).

Supplementary Figure 4

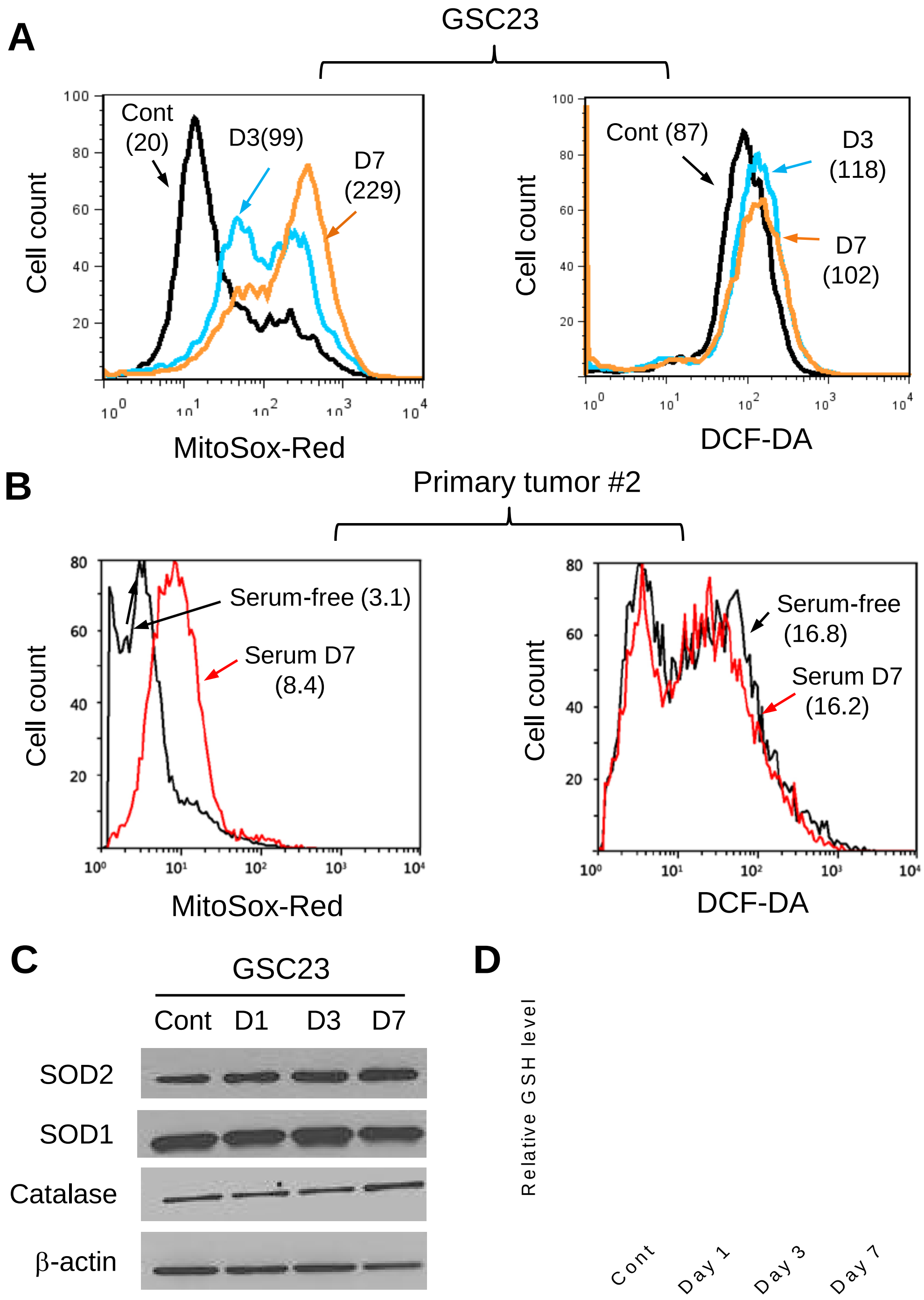

Fig S4. Effect of serum on mitochondrial superoxide, cellular ROS, and expression of antioxidant molecules in GSCs. (A) Effect of serum (5% FBS) on mitochondrial superoxide (measured by flow cytometry analysis with mitoSOX-Red staining) and total cellular ROS (measured by DC-FDA staining) in GSC23 cells. The numbers in parentheses indicate the median relative fluorescent intensity. (B) Effect of serum on mitochondrial superoxide and total cellular ROS in primary GBM cells (serum exposure for 7 days). (C) Western blot analysis of SOD1, SOD2, and Catalase in GSC23 cells before and after exposure to serum for 1-7 days. (D) Effect of serum on cellular glutathione (GSH) in GSC23 cells.

Supplementary Figure 5

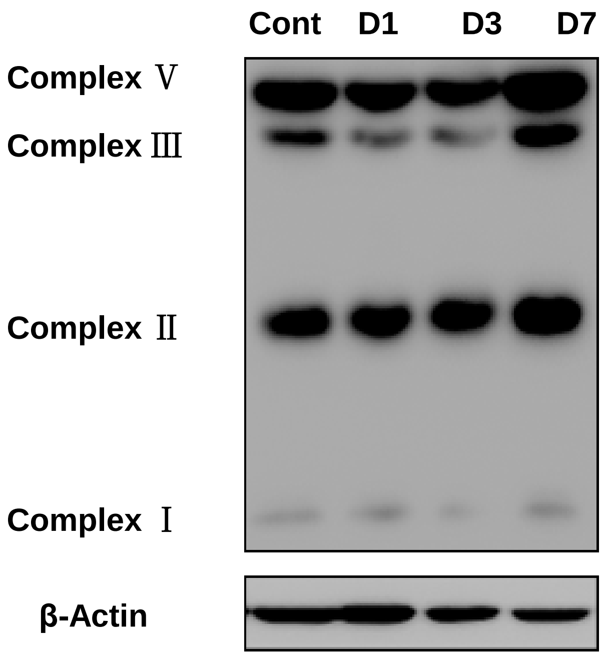

Fig S5. Effect of serum on the expression of mitochondria electron transport chain and the ATP synthase complexes. GSC11 cells were exposed to 5% FBS for 1, 3, and 7 days, and gene expression was analyzed by western blotting.

Supplementary Figure 6

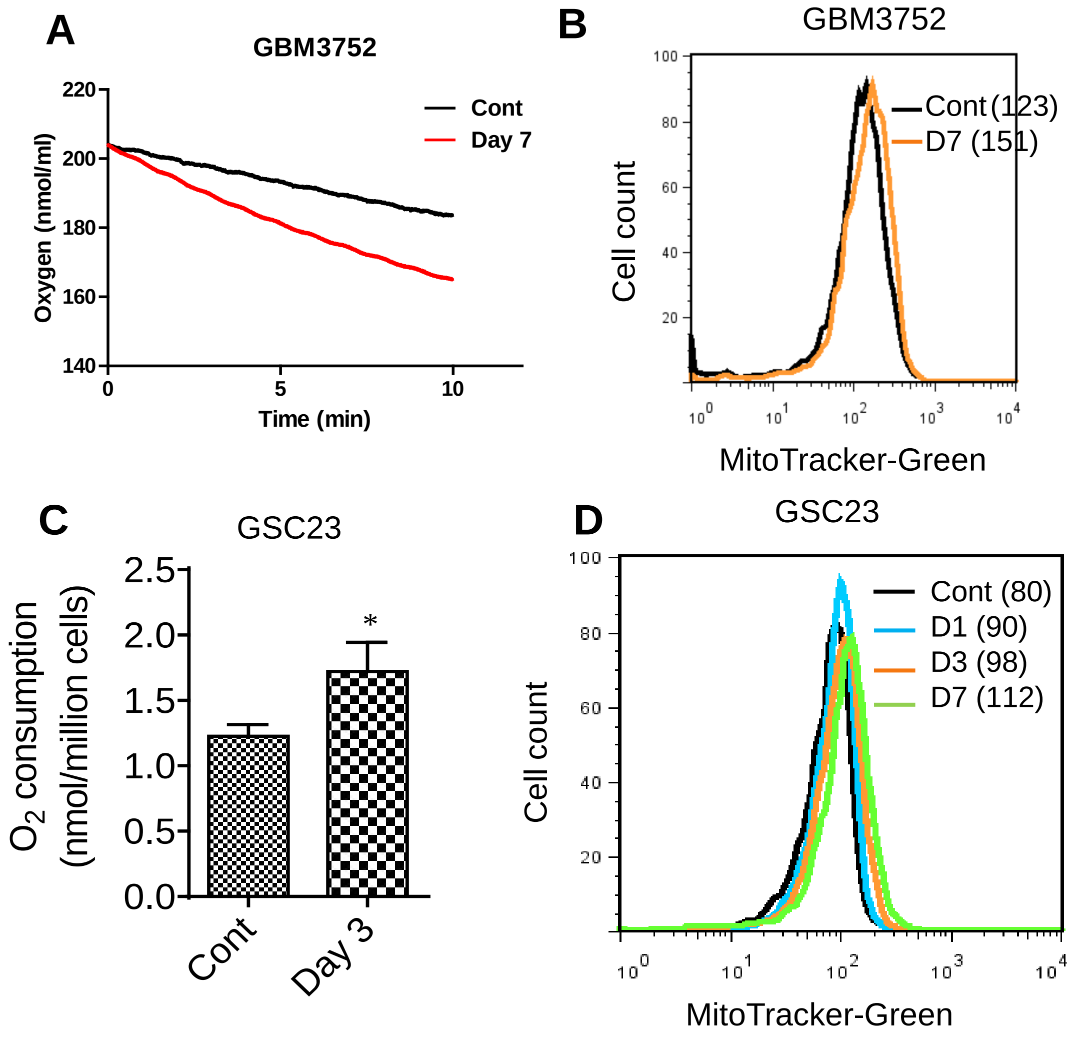

Fig S6. Activation of mitochondrial respiration by serum in GBM3752 and GSC23 cells. (A) Increase of oxygen consumption in GBM3752 cells after exposure to serum for 7 days. (B) No change in mitochondrial mass in GBM3752 cells exposed to serum for 7 days. The numbers in parentheses indicate the median fluorescent values. (C) Increase of oxygen consumption in GSC23 cells after exposure to serum for 3 days. *, $P<0.05$. (D) No change in mitochondrial mass in GSC23 cells exposed to serum for 1 day (D1), 3 days (D3), or 7days (D7) measured by MitoTraker-Green. The numbers in parentheses indicate the median fluorescent values.

Supplementary Figure 7

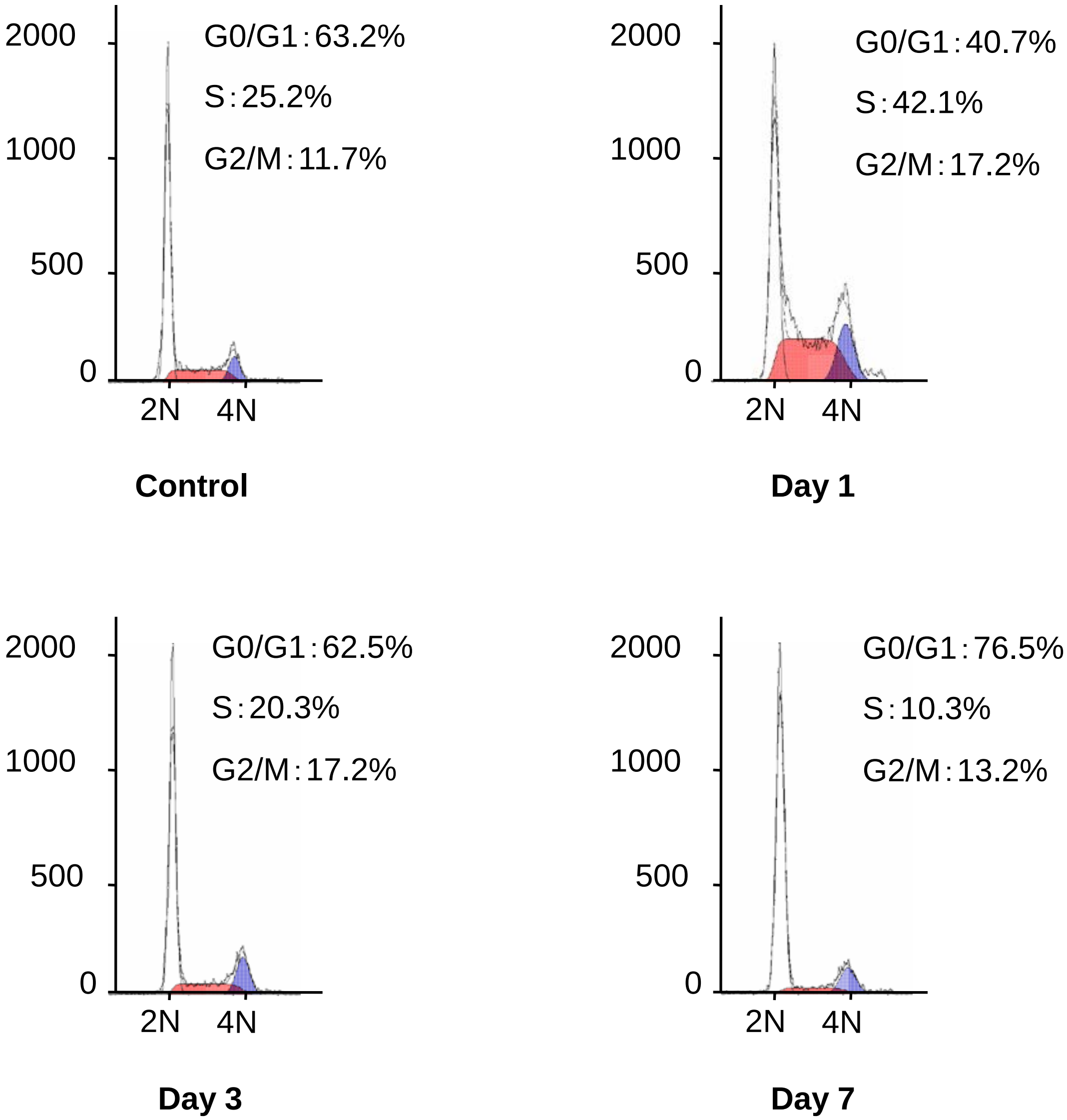

Fig S7. Effect of serum on cell cycle. GSC11 cells were exposed to 5% FBS for 1, 3, and 7 days, and cell cycle was analyzed by flow cytometry.

Supplementary Figure 8

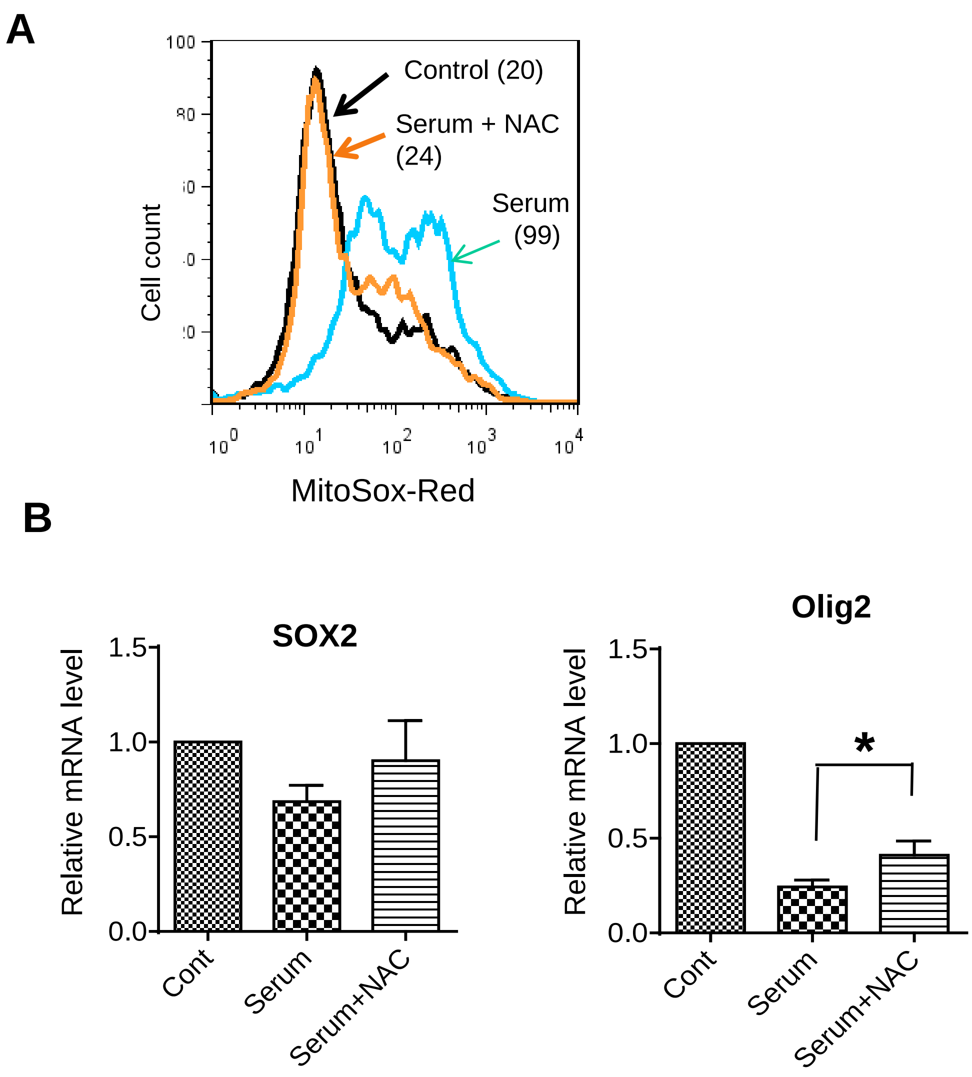

Fig S8. Effect of antioxidant N-acetylcysteine (NAC) on serum-induced changes in ROS and expression of SOX2 and Olig2. (A) Effect of NAC on mitochondrial superoxide levels in GSC23 cells exposed to serum. Cells were cultured with or without serum (5% FBS) in the presence or absence of 20 mM NAC for 3 or 7 days as indicated, and mitochondrial superoxide was measured using MitoSox Red. (B) NAC suppressed serum-induced loss of SOX2 and Olig2 expression. GSC23 cells were exposed to serum for 3 days in the presence or absence of 20 mM NAC as indicated, and the expression of SOX2 and Olig2 mRNA in each sample was measured by quantitative RT-PCR.
